# Challenges to implementing electronic trial data collection in primary care: a qualitative study

**DOI:** 10.1186/s12875-021-01498-6

**Published:** 2021-07-06

**Authors:** Christie Cabral, Kathryn Curtis, Vasa Curcin, Jesús Domínguez, Vibhore Prasad, Anne Schilder, Nicholas Turner, Scott Wilkes, Jodi Taylor, Sarah Gallagher, Paul Little, Brendan Delaney, Michael Moore, Alastair D. Hay, Jeremy Horwood

**Affiliations:** 1 Centre for Academic Primary Care, Bristol Medical School: Population Health Sciences, Canynge Hall, 39 Whatley Road, Bristol, BS8 2PS UK; 2 School of Population Health and Environmental Sciences, Faculty of Life Sciences and Medicine, King’s College London, Addison House 3.07, Guy’s Campus, London, SE1 1UL UK; 3 NIHR University College London Hospitals Biomedical Research Centre and evidENT, UCL Ear Institute, 91 Gower Street, London, WC1E 6AB UK; 4 Bristol Randomised Trial Collaboration (BRTC), Part of the Bristol Trial Centre, Bristol Medical School, University of Bristol, Canynge Hall, 39 Whatley Road, Bristol, BS82PS UK; 5 School of Medicine, Faculty of Health Sciences and Wellbeing, University of Sunderland, Sciences Complex, City Campus, Chester Road, Sunderland, SR1 3SD UK; 6 The Portland Practice, St Pauls Medical Centre, 121 Swindon Road, Cheltenham, GL50 4DP Gloucestershire UK; 7 Primary Care, Population Sciences and Medical Education, Faculty of Medicine, University Of Southampton, Southampton, SO17 1BJ UK; 8 Faculty of Medicine, Department of Surgery & Cancer, Imperial College London, South Kensington Campus, London, SW7 2AZ UK

**Keywords:** Primary health care, Electronic health records, Information technology

## Abstract

**Background:**

Within-consultation recruitment to primary care trials is challenging. Ensuring procedures are efficient and self-explanatory is the key to optimising recruitment. Trial recruitment software that integrates with the electronic health record to support and partially automate procedures is becoming more common. If it works well, such software can support greater participation and more efficient trial designs. An innovative electronic trial recruitment and outcomes software was designed to support recruitment to the Runny Ear randomised controlled trial, comparing topical, oral and delayed antibiotic treatment for acute otitis media with discharge in children. A qualitative evaluation investigated the views and experiences of primary care staff using this trial software.

**Methods:**

Staff were purposively sampled in relation to site, role and whether the practice successfully recruited patients. In-depth interviews were conducted using a flexible topic guide, audio recorded and transcribed. Data were analysed thematically.

**Results:**

Sixteen staff were interviewed, including GPs, practice managers, information technology (IT) leads and research staff. GPs wanted trial software that automatically captures patient data. However, the experience of getting the software to work within the limited and complex IT infrastructure of primary care was frustrating and time consuming. Installation was reliant on practice level IT expertise, which varied between practices. Although most had external IT support, this rarely included supported for research IT. Arrangements for approving new software varied across practices and often, but not always, required authorisation from Clinical Commissioning Groups.

**Conclusions:**

Primary care IT systems are not solely under the control of individual practices or CCGs or the National Health Service. Rather they are part of a complex system that spans all three and is influenced by semi-autonomous stakeholders operating at different levels. This led to time consuming and sometimes insurmountable barriers to installation at the practice level. These need to be addressed if software supporting efficient research in primary care is to become a reality.

**Supplementary Information:**

The online version contains supplementary material available at 10.1186/s12875-021-01498-6.

## Background

Within-consultation ‘hot’ recruitment of patients with incident conditions is significantly more challenging than ‘cold’ recruitment of patients with prevalent conditions, who can be contacted electronically or by letter [1]. The extra workload and time needed both to set up and to recruit within a normal consultation are major barriers to participation by GPs and practices [1–3]. Participation can be increased where there is perceived clinical value and / or benefit to patients, adequate remuneration for time and streamlined recruitment processes that minimize workload [1–4].

One approach to minimizing the workload and cost is to make use of data routinely collected with electronic health records (EHR) to identify eligible patients and collect outcome data [1]. ‘TRANSFoRm’ (Translational Research and Patient Safety in Europe) is an electronic trial data collection platform that integrates with the EHR to: perform automatic eligibility checking of entered Read/Snomed codes of patients upon presentation; capture electronic Case Report Form data part-filled from the EHR at pre-defined points in the study workflow; and use mobile and web portals to collect Patient Reported Outcome Measures (PROMs) [5]. TRANSFoRm was developed as part of a 5-year EU FP7 Programme. Following a successful pilot study in Poland [6], TRANSFoRm was used in the UK-based REST study, a 3-arm pragmatic trial of treatment for acute otitis media with discharge in children comparing topical, ‘immediate’ oral and ‘delayed’ antibiotic treatment options [7].

Otitis media with discharge (AOMd) is a painful and distressing condition and most children are treated in primary care with ‘immediate’ oral antibiotics [8, 9]. However, the use of systemic antibiotics risks side effects and antibiotic resistance [10, 11]. While responsible for a significant proportion of antibiotic prescribing, AOMd is not as common as infections such as tonsillitis and acute bronchitis, meaning individual GP practices would expect to recruit relatively small numbers of children. The infrequency of recruitment opportunities makes it more likely that potentially eligible children would be missed because clinicians will find it difficult to keep it in mind and to remember the process for recruitment. TRANSFoRm provided an automatic alert for potentially eligible children, guided and recorded recruitment procedure and auto-populated patient data. The Runny Ear STudy (REST) randomised controlled trial therefore aimed to recruit 175 GP practices across the United Kingdom and use TRANSFoRm to support efficient trial processes [7]. The results of the REST study will be reported elsewhere [12].

We conducted a nested qualitative study to describe the experience of primary care practice staff of REST trial processes. This paper reports the views and experiences of primary care staff deploying and/or using the TRANSFoRm trial software, within the context of UK primary care.

## Methods

Purposive sampling was used to select participants in order to capture maximum variation in views and experiences [13]. Primary care staff involved in trial processes were purposively sampled in relation to site, role and whether the practice was able to successfully recruit patients. Views were sought from recruiting clinicians, practice research staff, and those primary care staff involved in installing and supporting the TRANSFoRm software, which included staff in management and IT support roles.

In-depth interviews with primary care staff were conducted using a flexible topic guide to ensure that the primary issues are covered across all interviews, but enabled participants to introduce unanticipated issues [14]. The topic guide was revised to include new topic areas identified from earlier interviews, particularly around the barriers to implementing TRANSFoRm (supplementary file 1). The researcher used open-ended questioning techniques to elicit participants’ experiences and views of key events and participants were asked to provide examples. The interviews were conducted over the phone, lasted 20–45 min and were recorded using a digital voice recorder. Audio recordings were transcribed and anonymised to protect confidentiality.

Interview transcripts were imported into NVIVO 12 qualitative data analysis software. Analysis began shortly after data collection and was ongoing and iterative, informing further data collection and identifying changes needed to the topic guide. Thematic analysis [15], utilising a data-driven inductive approach, was used to identify and analyse patterns and themes of particular salience for participants and across the dataset using constant comparison techniques [16, 17]. A subset of transcripts was independently double coded by members of the team (CC and JH) and discussed to achieve coding consensus and maximal rigour. Sample size was informed by the concept of ‘information power’ [18], with analysis and sampling conducted in parallel and continuous assessment of the suitability of the information within the sample with regard to study objectives.

## Results

Sixteen primary care staff were interviewed: 9 GPs and 7 other staff, from recruiting and non-recruiting practices (including 1 practice that withdrew from the study) (Table 1). Some of the GPs had experience of recruiting to the trial and some had experience of getting the TRANSFoRm software working. Staff from recruiting practices had each recruited at least 1 child. All of the GPs were partners and their practice’s research leads, with years in practice ranging from 4 to 33. The non-clinical staff included practice managers, practice IT leads, a research coordinator and a research nurse (with no clinical role) who had experience of installing the TRANSFoRm software and the processes involved in getting it to work.

**Table 1:** Primary care staff qualitative interview sample

	Recruiting practices	Non-recruiting practices	Total
GPs	5	4	9
Non-clinical staff (practice managers, practice IT leads, research coordinator, research nurse)	2	5	7
Total	7	9	16

The findings are organised below into three thematic areas: views of automated data capture software; experiences of implementing new software; and the challenges to software implementation from the limited, varied and changing IT context in UK primary care.

### “*It puts the details in which is time saving*”: views of automated data capture

Participants were keen on the idea of a system that would automatically capture data on recruited patients. They faced considerable workload pressure, and this means there is little time for additional work, which sometimes was a barrier to participation in research studies. Research leads liked the idea of an automated system because they thought it could reduce the time taken for the research and therefore make it more possible for practices to participate.

“*I think as an idea it’s brilliant. … it means you haven’t got piles and piles of paperwork … that you’ve then got to somehow get scanned to email through. … it self-populates. … it puts the details in which is time saving. Cos time is one of the big things in general practice*” (GP09)

*“there’s not a spare minute in primary care at the moment. … Research in primary care, I think we’re, I think we’re struggling a bit. … There’s such a burden on, on GP time for major problems that research … the GPs would look at the studies and say, ‘Yeah, I don’t have time to do this’*.” (Research Nurse, ITA03)

### *“It has taken an awful lot of time”*: experiences of software

Participants felt that the software was not sufficiently developed for deployment in UK practices. GPs and non-clinical staff involved in installing TRANSFoRm and getting it to work described a long and frustrating process of trouble shooting and multiple reinstallations. Part of the software had to be installed individually on each recruiting clinician’s computer and limited access to these computers led to delays.

“*to be brutally honest with it was quite a nightmare … I’ve probably spent about 10 h in total trying to install the piece of software on one computer. Um, quite often there would be loads of errors with it installing, with it not working. Um, I’d then have to send emails to the people that were dealing with it. … it’s definitely taken so much longer than what we thought it was going to take.*” (Practice Operations Manager, ITA02)

*“the major problem, um, of the installation is actually getting time to get into the GP’s room. … we’re really limited on space so if that GP isn’t in there’ll be a locum in their room … from eight in the morning ‘til 6.30 or seven o’clock at night. So actually trying to get in to have… 2 h… is virtually impossible.* (Research Nurse, ITA03)

The biggest impact was the time taken to get TRANSFoRm installed and working in the already very time pressured context of primary care. This impacted on the GP practices other work, including preparing data for the Quality Outcomes Framework (QOF) – NHS performance management and payment of general practitioners. When the time commitment started to impede essential work, then practices started to consider withdrawing from the study.

*“I’ve spent literally hours on this trying to install the software, hours. I think our clinicians have said that, you know, enough’s enough. They don’t want me to spend any more time on it. … they [GP partners] just [got] cross ‘cause I wasn’t doing other things …. I do all the QOF stuff so all the quality registers and things and all the statistics, all the claims. … all that sort of was a bit on hold really.*” (Data Manager, IT01)

*“I started to refuse to install stuff because we were having so many problems with what it was doing to our computers … it was probably towards the end of the QOF year … I said I wasn’t prepared to put it back on until after we finished the QOF year because I couldn’t risk the machines not working. (GP09)*

Some participants reported financial costs to the practice as a result of participating in the study. At least one had paid for extra hours for their IT support person to try to get TRANSFoRm to work. Several participants felt that financial support to practices did not compensate for time spent on research activities for this study, with the relatively low number of expected recruits per practice and the high start-up demands of installation.

*“I’ve worked extra time to do it as well. I’ve, they’ve actually paid me extra to come in and do the REST software so I think that’s sort of annoyed them a little bit*.” (Data Manager, IT01)

### *“We’re not all IT proficient”*: variations in primary care IT provision and skills

The limited, varied and changing IT capacity across the primary care practices presented many challenges to installing and getting the new software to work.

At the practice level, there was limited staff with IT capacity and expertise. There were issues with varying versions of Windows and internet browsers and with the way in which GPs had individually adjusted settings on the EHR. There was the varied IT expertise and capacity in individual practices with many reliant on a GP, manager or administrator with only modest knowledge of IT. Windows admin rights (needed to install any software) were usually restricted to a small number of staff and not necessarily those with time or responsibility for setting up research studies. This meant that in many practices the person tasked with doing the work to get TRANSFoRm to function often struggled with the tasks and with understanding the various problems encountered.

“*we are a fairly small practice … it was pretty much … me on my own … just trying to go through the installation step by step to work out where it wasn’t working and then trying to work out why so, trial and error*” (GP06)

“*we didn’t locally have full admin rights. Well the Practice Manager did but you know, to get her to sit down for a couple of hours and set it all up was very difficult, she didn’t have a couple of hours.*” (Research Co-ordinator, ITA05)

There were issues with obtaining help from outsourced IT support. Practices all had some IT support provided by an external body, sometimes a private provider and sometimes a Commissioning Support Unit (CSU) or other NHS provider. CCGs commission local health services and NHS England the national services. Five different providers were mentioned by our relatively small number of participants. IT support arrangements varied with respect to whether the external body held exclusive admin rights for practice computers or supported research IT. Several practices reported that their external IT support provider would not assist because the software was not on the CCG approved list. When they were asked to provide support, these external bodies often raised concerns about the unknown TRANSFoRm software, were usually unfamiliar with software for research projects and slow in providing support due to limited capacity. In one very research active practice, the dedicated research co-ordinator described using her established good relationship with the external IT provider to obtain the support needed.

“*we’re not sort of in charge of our own IT, the IT goes out to another company … our IT people who are [name A], they’re not really supposed to give admin rights to anybody in a practice … [company name A]will not get involved with other people’s software. …they have a list of software that’s allowed on the system and if we’re going to put some other software onto it they will not support us installing that software”* (Practice Data Manager, ITA01)

“*we do have [name C] but I didn’t get them involved in it…’cause … [name C] wouldn’t help with it anyway. … because it hadn’t been signed off by our CCG so we shouldn’t be installing it on our computers*.” (Operations Manager, ITA02)

“*[name E] are our … IT support … they were much more obliging than I thought they'd be to be honest but we have worked with them before on other studies with software that downloads onto the PC so they were fine about it … we had a very good relationship with you know one particular person on the IT team, willing to help us*” (Research Co-ordinator, ITA05)

Some practices reported that they had to obtain permission from their CCG before installing software on their practice computers. The transition to Windows 10 during the trial was linked to the loss of practice level admin rights over computers in some CCG areas. Whether or not practices retained some admin rights over their computers (and therefore ability to install software) varied across recruited practices. When practices had to obtain permission from their CCG to install software, this could be a lengthy process. CCGs raised questions about the risk of this unknown software in terms of cybersecurity. The centralisation of management to the CCG was seen as supporting initiatives such as the single domain, which allows better sharing of patient notes between different types of practitioners in primary care. However, it also had the unintended consequence of restricting the installation of study specific software.

“*we had a big change at our practice erm, something called single domain which basically means that they’ve taken a lot of admin rights away from a lot of the users including me… cause I think it was becoming problematic across the practices that you know, we had free rein really. And that’s going to cause a problem with things like REST because we can’t install it, so you give us a set of instructions and we won’t be able to do it because it has to go to our localised IT who has to verify they're okay with it first.*” (Assistant Practice Manager, ITA04)

“*the CCG took it upon themselves to be responsible for all of our hardware and software, so when Windows 10 came for the whole of the CCG, they then took charge of everything really, which in a way makes sense because they paid for it and therefore they should control it and the flow of information that’s available and try to link it all up with other bits of the NHS, but as a result things … fell by the wayside, unfortunately.*” (GP05)

“*because of the way that the NHS is set up we had to get firewalls opened, … to enable the software to contact [trial database] and then also for them to contact back through to our software so basically you had to go through the firewall through a different port. … so that was quite complicated at the beginning, having to go through these firewalls by logging it with our IT and then our IT doing it and that took a while.*” (Data Manager, ITA01)

“*[I] installed the software once I had permission from the CCG and that took [from] July/August … until December … it’s just checking the security side of things, just make sure we’re not going to get any viruses... it’s about data protection, you know they want to make sure that no patient identifiable data is going to be sent over for the studies*.” (IT Support Manager, IT07)

CCG’s having control over practice software installation was reported by most practices but was not universal. A GP from one practice reported being able to install software freely. A GP from another practice, who had a role within the CCG, was able to use their influence to get the software installed in his practice.

“*I think [the CCG] are quite – lax might be the wrong word, but we can install software and we do install software. So we’ve installed software for other research studies with no problems*.” (GP09)

“*[we] needed to change our operating system … [to] Windows 10 … we couldn’t [install] ourselves any more, we had to get the CCG computer boffins in to do it for us, they didn’t want to do it because they said its software may corrupt the NHS software and they wanted more assurance from higher levels than me that it was all safe to go, soooo… I got cross … told them it was all, it had been approved at high level, … co-ordinated at committee level and approved and was being used elsewhere and they shouldn’t be so silly … so they then did come and put it on for me, so it’s now up and running*” (GP05)

## Discussion

### Summary

Research leads in participating practices supported the idea of software that would streamline and automate study processes. However, it was very time consuming to deploy TRANSFoRm software and configuring PCs at practice level to allow the software to run as intended, involving clinical and non-clinical staff, and sometimes outsourced IT support. Some practices reported multiple problems with software deployment, in a few cases leading to practices withdrawing from the trial before recruiting a single patient. Primary care staff described multiple challenges that derived from the limited, varied and changing IT capacity within primary care practices.

### Strengths and limitations

The qualitative interviews captured a range of views from clinicians and other primary care staff involved in the study. It was possible to purposively sample for clinicians and non-clinicians from recruiting and non-recruiting practices, which captured a good range of views and experiences with respect to the trial. The primary care staff also described considerable variety in terms of the IT arrangements for practices, although it seems likely that this study does not capture the full range of variation in UK general practice IT arrangements. Recruitment was cut short by the Covid-19 pandemic. However, sufficient information power [18] was reached for the core themes presented.

### Comparison with existing literature

Implementing new technology in health services is inherently challenging [19]. Issues such insufficiently tested software and organisations that are inadequately set up for installing and validating new software, are common barriers to new technologies in health care [19, 20]. Complex and varied settings also make it difficult to adequately test software, because settings can affect function in unpredictable ways [21]. The more complex a setting into which new technology is introduced, the less likely it is to be adopted and sustained [22, 23]. The findings of this study unpacked some of this complexity and identified contributing issues at practice level, in the interaction between practices and CCG, and from NHS level policies on IT infrastructure. These are key issues that need to be considered when developing software designed to facilitate efficient and pragmatic trials in primary care.

At a practice level, the internal and external IT support was not sufficient for the challenges posed by the introduction of this new trial data collection software. This resulted in the considerable workload of installation and troubleshooting falling on individual practices. The limited IT support resulted from a combination of practice level decisions about resourcing and the NHS Primary Care Digital Services Operating Model that specifies CCG provided IT support and makes no mention of research studies [24]. This type of organisational complexity, where decisions are made at different levels by autonomous but interrelated bodies (here GP practices, CCGs and sometimes external IT support providers), poses a substantial challenge to the implementation of innovative software [23, 25]. Considerable technical support is needed to implement new software across the varied contexts of multiple practices and CCGs, this is not available at practice or CCG level and is unlikely to be affordable for an individual research study.

Changes in practice software and support arrangements, driven by NHS IT service improvement initiatives, were another key barrier to the implementation of new research software on practice computers. Health systems are complex and constantly changing in response to internal and external drivers and the pace of change is yet another challenge faced when implementing new software [19, 22, 26]. At the time of this study there was no national guidance for CCGs from NHS X or NHS Digital as to what assessment was required and national assurance process for new software. During the REST trial, many practices updated their operating systems and administrative rights over software installation transferred from individual practices to CCGs,. These changes were driven by NHS initiatives to improve IT service provision and align GP IT operating arrangements, which include adherence to GDPR and measures to protect from ransomware attacks [24]. However, these changes had the unintended consequence of forming additional barriers to the implementation of software that had to be installed at a practice level and was designed to extract and export (anonymised) patient data from EHR and to a secure server outside the NHS. Primary care IT systems are not solely under the control of individual practices or CCGs or the NHS. Rather they are part of a complex adaptive system that spans all three and includes other stakeholders, such as the EHR software providers, all of which may drive different types of change. Any new software needs to be able to operate within this complex adaptive system and successful implementation requires engagement with all the key stakeholders.

## Conclusions

Pragmatic trials, which need to recruit within consultations, are essential for the production of high-quality evidence about what works under normal clinical care [1]. Primary care is a busy and time pressured environment [2, 3]. Software that links to EHR and automates some of the trial processes and data collection could support greater participation and more efficient trial designs [1, 5]. In order for that to become a reality, there is need for: 1) higher priority placed on research IT by all the stakeholders who influence primary care IT provision; 2) provision of substantial technical support to GP practices to get any new software functioning smoothly without adding to practice workload; and 3) development and testing of software like TRANSFoRm as a platform service to ensure that it is deployed and running smoothly before it can be used with live clinical trials. The UK NHS recently announced the GP IT Futures framework [27], from the new Digital Care Services model, to supply IT systems and services to GP practices and this may offer better opportunities for engagement by developers of health research software. Although individual projects may be able to work towards this objective [1, 5, 7], the scale of the challenge may require structural changes in NHS IT provision to support efficient data collection initiatives as part of the core digital services provision, not as an ad-hoc bolt on.

## Supplementary Information

**Additional file 1.** Topic Guide.

## Data Availability

The data that support the findings are not publicly available because it is difficult to fully anonymise interview data and participants from the same primary care practice may be able to identify each other. Data are however available from the authors upon reasonable request and with permission of the University of Bristol and the South Central Oxford B Research Ethics Committee.

## Abbreviations

CCG: Clinical Commissioning Group
CSU: Commissioning Support Unit
HER: Electronic health record
GDPR: General Data Protection Regulation
GP: General practitioner
IT: Information technology
NHS: National Health Service
NHS X: National Health Service Digital Transformation Unit
AOMd: Otitis media with discharge
PC: Personal computer
PROMs: Patient Reported Outcome Measures
QOF: Quality Outcomes Framework
REST: The Runny Ear STudy
TRANSFoRm: Translational Research and Patient Safety in Europe

## References

[1] van Staa T-P, Dyson L, McCann G, Padmanabhan S, Belatri R, Goldacre B (2014). The opportunities and challenges of pragmatic point-of-care randomised trials using routinely collected electronic records: evaluations of two exemplar trials. Health Technol Assess.

[2] Brodaty H, Gibson LH, Waine ML, Shell AM, Lilian R, Pond CD (2013). Research in general practice: a survey of incentives and disincentives for research participation. Ment Health Fam Med.

[3] Salmon P, Peters S, Rogers A, Gask L, Clifford R, Iredale W (2007). Peering through the barriers in GPs’ explanations for declining to participate in research: the role of professional autonomy and the economy of time. Fam Pract.

[4] Ngune I, Jiwa M, Dadich A, Lotriet J, Sriram D (2012). Effective recruitment strategies in primary care research: a systematic review. Qual Prim Care.

[5] Delaney BC, Curcin V, Andreasson A, Arvanitis TN, Bastiaens H, Corrigan D (2015). Translational medicine and patient safety in Europe: TRANSFoRm—architecture for the learning health system in Europe. BioMed Res Int.

[6] Ethier J-F, Curcin V, McGilchrist MM, Choi Keung SNL, Zhao L, Andreasson A (2017). eSource for clinical trials: Implementation and evaluation of a standards-based approach in a real world trial. Int J Med Inform.

[7] Curtis K, Moore M, Cabral C, Curcin V, Horwood J, Morris R (2020). A multi-centre, pragmatic, three-arm, individually randomised, non-inferiority, open trial to compare immediate orally administered, immediate topically administered or delayed orally administered antibiotics for acute otitis media with discharge in children: The Runny Ear Study (REST): study protocol. Trials.

[8] Smith L, Ewings P, Smith C, Thompson M, Harnden A, Mant D (2010). Ear discharge in children presenting with acute otitis media: observational study from UK general practice. Br J Gen Pract.

[9] Williamson I, Benge S, Mullee M, Little P (2006). Consultations for middle ear disease, antibiotic prescribing and risk factors for reattendance: a case-linked cohort study. Br J Gen Pract.

[10] Costelloe C, Metcalfe C, Lovering A, Mant D, Hay AD (2010). Effect of antibiotic prescribing in primary care on antimicrobial resistance in individual patients: systematic review and meta-analysis. BMJ.

[11] Goossens H, Ferech M, Vander Stichele R, Elseviers M (2005). Outpatient antibiotic use in Europe and association with resistance: a cross-national database study. Lancet.

[12] Hay AD, Curtis K, Taylor J, Harris S, Rowley K, Sadoo A, et al. The Runny Ear Study: immediate oral, immediate topical or delayed oral antibiotics for acute otitis media with discharge. The REST randomised controlled trial. UK: NIHR Health Technology Assessment; 2022. https://www.journalslibrary.nihr.ac.uk/programmes/hta/168501.

[13] Sandelowski M (1995). Sample size in qualitative research. Res Nurs Health.

[14] Britten N (1995). Qualitative interviews in medical research. BMJ.

[15] Braun V, Clarke V (2006). Using thematic analysis in psychology. Qual Res Psychol.

[16] Boyatzis R (1998). Transforming qualitative information: thematic analysis and code development.

[17] Charmay K (2006). Constructing grounded theory: a practical guide through qualitative analysis.

[18] Malterud K, Siersma VD, Guassora AD (2016). Sample size in qualitative interview studies: guided by information power. Qual Health Res.

[19] Greenhalgh T, Wherton J, Papoutsi C, Lynch J, Hughes G, Hinder S (2017). Beyond adoption: a new framework for theorizing and evaluating nonadoption, abandonment, and challenges to the scale-up, spread, and sustainability of health and care technologies. J Med Internet Res.

[20] Gentil M-L, Cuggia M, Fiquet L, Hagenbourger C, Le Berre T, Banâtre A (2017). Factors influencing the development of primary care data collection projects from electronic health records: a systematic review of the literature. BMC Med Inform Decis Mak.

[21] Abbott PA, Foster J, de Fatima MH, Dykes PC (2014). Complexity and the science of implementation in health IT—Knowledge gaps and future visions. Int J Med Inform.

[22] Atun R, de Jongh T, Secci F, Ohiri K, Adeyi O (2010). Integration of targeted health interventions into health systems: a conceptual framework for analysis. Health Policy Plann.

[23] Cresswell K, Sheikh A (2013). Organizational issues in the implementation and adoption of health information technology innovations: an interpretative review. Int J Med Inform.

[24] NHS (2016). Securing excellence in primary care (GP) digital services: the primary care (GP) digital services operating model 2019–2021.

[25] Sligo J, Gauld R, Roberts V, Villa L (2017). A literature review for large-scale health information system project planning, implementation and evaluation. Int J Med Inform.

[26] de Lusignan S, van Weel C (2006). The use of routinely collected computer data for research in primary care: opportunities and challenges. Fam Pract.

[27] NHS Digital (2020). Future GP IT systems and services.

