# Supplementary material for: Challenges to implementing electronic trial data collection in primary care: a qualitative study
Source: BMC Fam Pract. 2021 Jul 6;22:147. doi: 10.1186/s12875-021-01498-6 (PMC8259773; doi:10.1186/s12875-021-01498-6)
Supplement: Supplementary file 1 — Additional file 1. Topic Guide. [file 12875_2021_1498_MOESM1_ESM.pdf]

The Runny Ear STudy (REST)

Staff Interview Topic Guide

1. Introduction, background & verbal consent

- Thanks, introduce self, re-state purpose of the interview, structure
- Discussion of how interview will be recorded, right to withdrawal, issues of confidentiality, anonymisation and informed consent. – *if agree start audio recorder*
- Verbal consent
    1. You agree to our conversation being audio recorded?
    2. You know you are free to stop the interview at any point and you may skip questions you would prefer not to answer?
    3. You understand that quotations from the interview may be used to illustrate our findings but it will not be possible to trace who said them?
    4. You understand that we will keep a written record of the interview but without anything that could identify you for future research?
- Background information on participant (job title, length of time practising, special interests)

2. Treatment views (clinicians only)

- Usual practice for child's ear pain before REST?
    - Any use of Ciprofloxacin ear drops or delayed oral antibiotics?
- Views of 3 treatment options
    - barriers, facilitators and adherence?
- Change in views of treatment since starting the trial

3. Experience of REST Trial (all staff)

- Views of being asked to take part in the *REST* study?
- Experiences of REST trial software implementation
    - Probe around:
        - Practice hardware / software issues (Windows version, admin rights)
        - IT support
        - Trial software issues (pop-up, meaning of messages)
        - CCG permissions, NHS firewall
- Did you take part in recruit of any participants?
    - If yes: How easy did you find it to recruit participants into the REST Trial?
        - Probe around:
            - Clinician factors (e.g. time constraints)
            - Parent factors (any reasons given for declining?)
    - Parental/patients reaction to ear drops & delayed oral antibiotics - acceptability

4. Views of potential for change in practice (clinicians only)

- If Ciprofloxacin ear drops or delayed oral antibiotics were to shown to be better than immediate oral antibiotics, do you think that it would change practice?
    - Why or why not?
    - Barriers and facilitators to change
    - Implications?
- Are there any changes that would need to be implemented for it to be rolled out to standard care? (information/support needs, guidance, training)

5. Any other issues

- Any other issues the participant would like to raise?

Thank them for their time.
